# Dexmedetomidine Inhibits TLR4/NF-κB Activation and Reduces Acute Kidney Injury after Orthotopic Autologous Liver Transplantation in Rats

**DOI:** 10.1038/srep16849

**Published:** 2015-11-20

**Authors:** Hui Yao, Xinjin Chi, Yi Jin, Yiheng Wang, Pinjie Huang, Shan Wu, Zhengyuan Xia, Jun Cai

**Affiliations:** 1 The Third Affiliated Hospital of Sun Yat-sen University, Department of Anesthesiology, Guangzhou, 510630, China; 2 The Third Affiliated Hospital of Sun Yat-sen University, Department of Pathology, Guangzhou, 510630, China; 3 The First Affiliated Hospital, University of South China, Department of Anesthesiology, Hengyang, 421001, China; 4 Li Ka Shing Faculty of Medicine, University of Hong Kong, Department of Anesthesiology, 21 Sassoon Road, Hong Kong SAR, China

## Abstract

Patients who undergo orthotopic liver transplantation often sustain acute kidney injury(AKI). The toll-like receptor 4(TLR4)/Nuclear factor-кB(NF-кB) pathway plays a role in AKI. Dexmedetomidine(Dex) has been shown to attenuate AKI. The current study aimed to determine whether liver transplantation-induced AKI is associated with inflammatory response, and to assess the effects of dexmedetomidine pretreatment on kidneys in rats following orthotopic autologous liver transplantation(OALT). Seventy-seven adult male rats were randomized into 11 groups. Kidney tissue histopathology and levels of blood urea nitrogen(BUN) and serum creatinine(SCr) were evaluated. Levels of TLR4, NF-κB, tumor necrosis factor-α, and interleukin-1β levels were measured in kidney tissues. OALT resulted in significant kidney functional impairment and tissue injury. Pre-treatment with dexmedetomidine decreased BUN and SCr levels and reduced kidney pathological injury, TLR4 expression, translocation of NF-κB, and cytokine production. The effects of dexmedetomidine were reversed by pre-treatment with atipamezole and BRL44408, but not ARC239. These results were confirmed by using α_2A_-adrenergic receptor siRNA which reversed the protective effect of dexmedetomidine on attenuating NRK-52E cells injury induced by hypoxia reoxygenation. In conclusion, Dexmedetomidine-pretreatment attenuates OALT-induced AKI in rats which may be contributable to its inhibition of TLR4/MyD88/NF-κB pathway activation. The renoprotective effects are related to α_2A_-adrenergic receptor subtypes.

Orthotopic liver transplantation (OLT) is the most effective therapy for end stage liver disease^1^. However, postoperative acute kidney injury (AKI) is a common and severe complication following OLT. Approximately 30–50% of patients undergoing liver transplantation have reportedly developed AKI^2^, which could adversely affect patient survival. Mechanisms that contribute to this complication remain unclear^3^. In addition, there are no effective and preventative treatment strategies to combat AKI following OLT. Therefore, there is a need to explore underlying mechanisms of AKI, and develop effective strategies for renal protection.

There are many causes of AKI following OLT, which involve multiple factors. Among these factors, renal ischemia–reperfusion injury (IRI) caused by perioperative renal hypoperfusion is considered as one of the most important independent risk factors^4^,^5^,^6^. The specific mechanism of renal IRI remains unclear. However, many theories have been proposed. Recent reports have indicated that ischemia-reperfusion (I/R) is associated with an inflammatory cascade and polymorphonuclear neutrophil (PMN) activation^7^,^8^. Endothelial injury and dysfunction following renal ischemia has been shown to result in large releases of inflammatory mediators and adhesion molecules such as interleukin (IL)-1, IL-6, IL-8, tumor necrosis factor (TNF)-α, P-selectin, E-selectin, intercellular adhesion molecule (ICAM)-1, etc. These cytokines induce tubular epithelial cell necrosis and renal tubular atrophy^9^. Recent studies have demonstrated that the toll-like receptor (TLR4)/Nuclear factor-кB (NF-кB) pathway plays a dominant role in mediating deleterious effects in renal IRI^10^,^11^,^12^. Several studies^13^ have demonstrated that TLR4 expressions increased in renal tubular epithelial cells after renal ischemia.

Dexmedetomidine (Dex) is a highly selective agonist of α2-adrenergic receptors, which is widely used in clinical anesthesia^14^. Beneficial effects of Dex include effective sedation, analgesia, hemodynamic stabilization, as well as anti-inflammatory and diuretic effects without respiratory depression and drug-dependency issues^15^. In addition, Dex has been shown to ameliorate IRI in several organs including brain, heart and kidney through anti-inflammatory, anti-apoptotic or anti-oxidative effects^16^,^17^,^18^,^19^. Several studies have demonstrated that dexmedetomidine may inhibit inflammatory mediator levels including IL-1, IL-6 and TNF-α^20^,^21^,^22^. However, few studies have been conducted on the mechanism of its anti-inflammatory effects. Therefore, this study aims to observe the renal protective effects of Dex to explore its relevant mechanisms, and to determine which adrenoceptor subtype mediates this effect.

## Results

### General parameters

There was no significant difference in weight and time of anhepatic phase between any of the groups (*P* > 0.05). (Table 1)

### Dex, atipamezole, BRL44408 and ARC239 had no effect on normal kidney function and histology

Rats treated with Dex, atipamezole, BRL44408 or ARC239 without OALT all revealed no significant changes in blood urea nitrogen (BUN) and serum creatinine (SCr) levels compared with the S group. Dex, atipamezole, BRL44408 and ARC239 also had no effect on morphology and histological scores of normal kidneys.

### Dexmedetomidine reduced remote kidney function impairment in a dose-dependent manner

**Figure 1:**
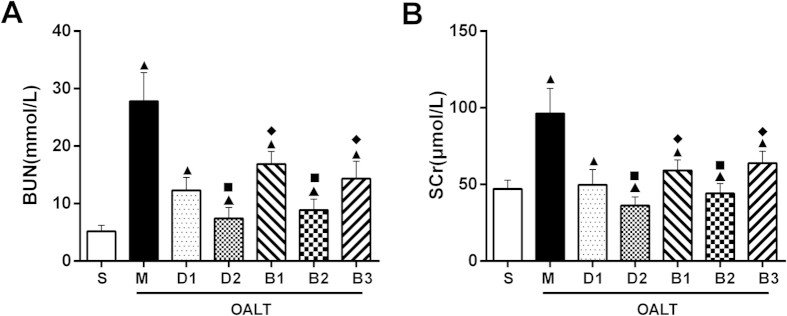
Alterations in (A) plasma urea and (B) plasma creatinine concentrations during OALT with the presence of dexmedetomidine (10 μg/kg for the D1 group and 50 μg/kg for the D2 group). Data are represented as mean ± standard deviation, *n* = 7. ^▲^*P* < 0.01 *vs.* S group, ^■^*P* < 0.01 *vs.* M group, and ^♦^*P* < 0.01 *vs.* D2 group.

Rats that underwent OALT-induced AKI (M group) exhibited significant kidney damage as reflected by the significant increase in BUN and SCr concentrations, compared with sham-operated rats (S group) (*P* < 0.05, Fig. 1A,B).

Compared with the M group, BUN and SCr levels were significantly reduced in groups pre-treated with dexmedetomidine, especially in D2 group with high doses of dexmedetomidine (*P* < 0.05); D1 group (*P* < 0.05) and D2 group (*P* < 0.05) (Fig. 1A,B).

### Dexmedetomidine dose-dependently attenuates I/R-mediated renal pathological injury

**Figure 2:**
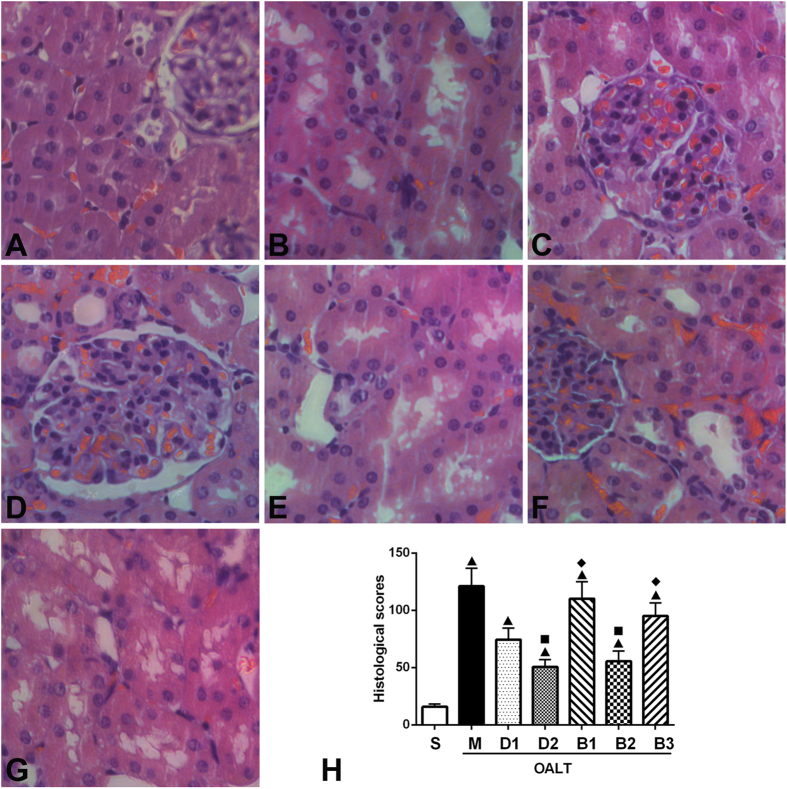
Histological analysis of tubulointerstitial injury and histological scores of kidneys. (**A**) S group, (**B**) M group, (**C**) D1 group, (**D**) D2 group, (**E**) B1 group, (**F**) B2 group, (**G**) B2 group, and (**H**) histological scores of various groups. Data are represented as means ± standard deviation, *n* = 7. ^▲^*P* < 0.01 *vs.* S group, ^■^*P* < 0.01 *vs.* M group, and ^♦^*P* < 0.01 *vs.* D2 group.

Kidneys taken from sham-operated rats (Fig. 2A) had normal tubular histology. Rats that underwent OALT demonstrated severe acute tubular damage. These features included tubular cell swelling, tubular dilatation and nuclear condensation (Fig. 2B). Dexmedetomidine decreased I/R-induced tubular damage (Fig. 2C,D).

Histological scores of rats in the M group were significantly higher than S group (*P* < 0.05). Histological scores significantly decreased in D1 and D2 groups, especially in D2 group, compared with scores in the M group (*P* < 0.05). However, these scores were still higher than S group (*P* < 0.05) (Fig. 2H).

### Dexmedetomidine reduces proinflammatory cytokine generation in kidney tissues of rats following OALT

**Figure 3:**
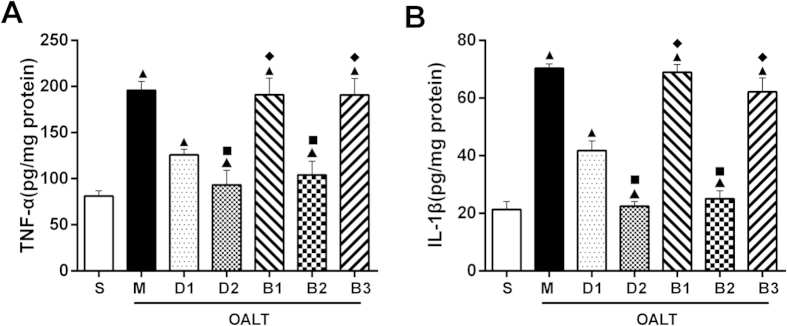
Effects of dexmedetomidine on OALT-induced upregulation of TNF-α and IL-1β levels in kidney tissue. Kidney tissues samples were taken at eight hours after liver reperfusion. Data are represented as means ± standard deviation, *n* = 7. ^▲^*P* < 0.01 *vs.* S group, ^■^*P* < 0.01 *vs.* M group, and ^♦^*P* < 0.01 *vs.* D2 group.

ELISA assays were performed to evaluate I/R-induced changes in TNF-α and IL-1β levels in kidneys, and to determine whether pre-treatment with dexmedetomidine could reduce inflammation. Results presented here revealed that TNF-α levels in the M group significantly increased, compared with the S group (*P* < 0.05, Fig. 3A). Pre-treatment with Dex in D1 and D2 groups significantly inhibited IRI-induced upregulation of TNF-α production (*P* < 0.05, Fig. 3A). Our results also revealed that TNF-α levels were inhibited by dexmedetomidine in a dose-dependent manner. Proinflammatory cytokine levels were lower in the D2 group (50 μg/kg of Dex) than in the D1 group (10 μg/kg of Dex) (*P* > 0.05, Fig. 3A). Similar results were observed for IL-1β levels (*P* < *0.05*, Fig. 3B).

### Dexmedetomidine regulates OALT-induced inflammatory responses by blocking TLR4/NF-κB pathway activation

**Figure 4:**
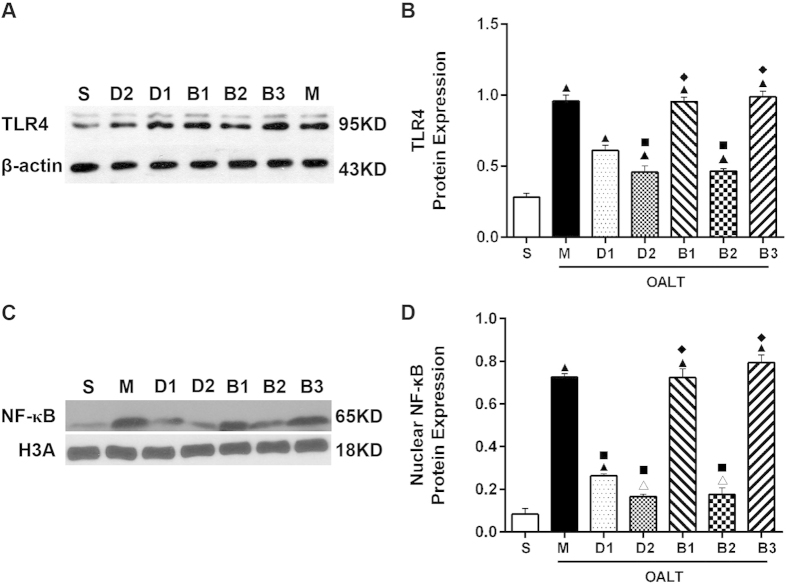
Dexmedetomidine downregulated TLR4 and NF-кB protein expressions after OALT: (**A**) representative Western blotting for TLR4 in total proteins, (**B**) densitometric analysis of TLR4 protein expressions were normalized to β-actin content; (**C**) representative Western blotting for NF-кB P65 in nuclear proteins, (**D**) densitometric analysis of NF-кB P65 protein expressions were normalized to H3A content; and data are represented as means ± standard deviation. ^▲^*P* < 0.01, ^△^*P* < 0.05 *vs.* S group; ^■^*P* < 0.01, ^▢^*P* < 0.05 *vs.* M group; ^◆^*P* < 0.01, ^◇^*P* < 0.05 *vs.* D2 group.

Since TLR4/NF-κB/p65 pathways have been shown to be closely associated with inflammation, we investigated whether dexmedetomidine exerts its effects through these signaling pathways. Western blotting revealed that TLR4 protein was low expression in the S group. TLR4 expression in renal tissues in the M group was significantly higher than S group (*P* < 0.05, Fig. 4A). I/R-induced TLR4 expressions were significantly inhibited in D1 and D2 groups (*P* < 0.05, Fig. 4B).

**Figure 5:**
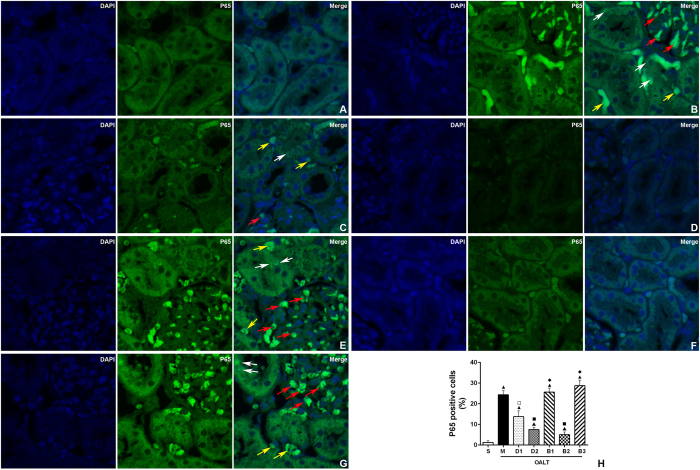
Representative confocal microscopic images shows the localization of NF-κB p65 by indirect immunofluorescence staining. (**A**) S group; (**B**) M group; (**C**) D1 group; (**D**) D2 group; (**E**) B1 group; (**F**) B2 group; (**G**) B3 group and (**H**). The proportion of P65 positive cells in tubular epithelial cells or glomerular cells. The expression of P65 in kidney tissues of different groups was detected by immunofluorescence staining under a laser scanning confocal microscope. Blue corresponds to nuclear staining and green corresponds to NF-κB p65 staining. Original magnification under ×400. Red arrows indicate P65 positive glomerular cells, white arrow indicate P65 positive tubular epithelial cells, and yellow arrow points at interstitial cells.

Immunofluorescence-staining and western blotting demonstrated that NF-κB was mainly in the nuclei of tubules of the renal cortex in the M group (*P* < 0.05, Figs 4C,D and 5). Immunofluorescence and western blotting results revealed that nuclear translocation levels of NF-κB in the M group were significantly up-regulated, compared with the S group (*P* < 0.05, Figs 4C,D and 5); and dexmedetomidine significantly decreased NF-κB activation induced by OALT (*P* < 0.05, Figs 4C,D and 5).

### Dexmedetomidine attenuats renal injury of the rats following OALT by activation of the a2A adrenergic receptor subtype

#### Effects of α_2-_adrenoceptor antagonists on decrease in I/R-induced renal injury by dexmedetomidine

As previously shown, dexmedetomidine significantly attenuated kidney damage which manifested as the significant decrease in BUN and SCr concentrations (Fig. 1A,B). Moreover, both atipamezole (a non-selective α_2_-antagonist) and BRL44408 (a α_2A_-subtype adrenoceptor antagonist) reversed the protective effect of dexmedetomidine on renal injury. However, ARC239 (a α_2B_-subtype adrenoceptor antagonist) did not block the protective effect of dexmedetomidine on kidneys (Fig. 1A,B).

Similar results were observed in histological analyses of tubulointerstitial injuries and histological scores of kidneys. Scores in B1 and B3 groups were higher than D2 group (*P* < 0.05, Fig. 2). However, scores in the B2 group were similar to scores in the D2 group (*P* > 0.05, Fig. 2).

#### Effects of α_2_-adrenoceptor antagonists on dexmedetomidine -induced inhibition of TLR4/NF-κB-inflammatory factor signaling pathway activation caused by OALT

As shown above, TLR4/NF-κB-inflammatory factor signal pathway was activated following OALT (M group). However, this phenomenon was attenuated by dexmedetomidine (D1 and D2 groups). In addition, the effect of dexmedetomidine was reversed by atipamezole and BRL44408, but not by ARC239. (Figs 3,4 and 5)

#### α_2A_-adrenergic receptor knockdown reversed the protection effects of dexmedetomidine on preventing NRK-52E cells from HR injury

**Figure 6:**
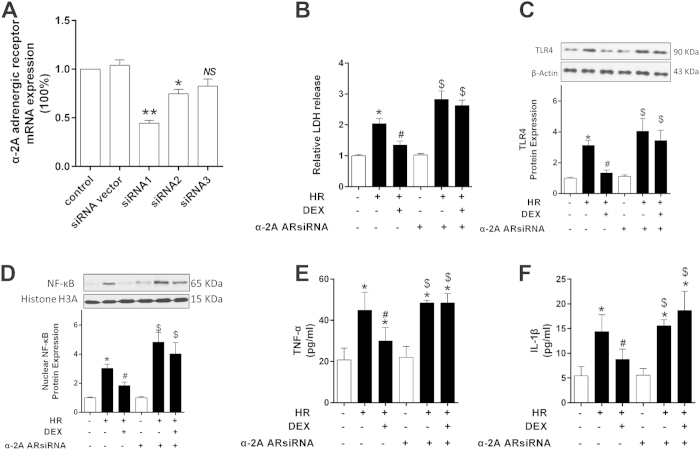
Effects of Dex (1 nM) and knockdown of α-2A adrenergic receptor on H/R-induced cell damage in NRK52E cells. (**A**) Determine of specific α-2A adrenergic receptor siRNA cassettes. (**B**) Relative lactate dehydrogenase (LDH) release of NRK-52E cells. (**C**) Determine of TLR4 expression by western blot. (**D**) Determine of nuclear NF-κB expression by western blot. (**E**) TNF-α release of NRK-52E cells. (**F**) IL-1β release of NRK-52E cells. Bars are mean ± standard deviation from four independent experiments. *P < *0.05* vs. control group; ^*#*^*P* < *0.05* vs. H/R group; ^*$*^*P* < *0.05* vs. HR + DEX group.

siRNA administration, as a gradually mature technology, was used in our current study to knock down α_2A_-adrenergic receptor(AR) induction. First, in NRK-52E cell line, we tested three α_2A_-AR siRNA sequences and determined that sequence 1 was the most effective in inhibiting α_2A_-AR mRNA expression (Fig. 6A). Then, we confirmed that effective knock down of α_2A_-AR expression in NRK-52E cells exposed to H/R and/or Dex. α_2A_-AR siRNA significantly increased cell damage following H/R, evidenced by increased LDH release (Fig. 6B). Next, TLR4, NF-κB p65 and cytokines(TNF-α and IL-1β) as important indexes of TLR4/NF-κB pathway, were assessed to verify the effects of Dex on TLR4/NF-κB signaling transduction. As shown in Fig. 6C–F, TLR4, NF-κB p65 nuclear translocation and cytokines was significantly increased in H/R group (*P* < 0.05 vs. sham) while significantly decreased with Dex treatment (*P* < *0.05*). However, this protective effect of Dex was reversed by α_2A_-AR knock down by siRNA, evidenced by the higher expression of TLR4 and NF-κB, and more LDH and cytokines release in the H/R +α_2A_-AR siRNA + Dex group than the H/R + Dex group (*P* < *0.05*, Fig. 6C–F).

## Discussion

OLT-AKI is one of the most important factors that affect prognosis, and often results in increased morbidity. However, its specific mechanism remains unclear^23^. The pathophysiology of OLT is complex; which includes perioperative hypotension^24^, as well as occlusion of the portal vein (PV) and inferior vena cava (IVC), resulting in renal I/R^42^,^5^. This is considered as one of the most important independent risk factors of AKI. In addition, cyanotic kidney and intestinal endotoxemia induced by occlusion of IVC and remote kidney damage induced by liver transplantation^25^ could give rise to OLT-induced AKI^26^.

The current rat model closely simulated most procedures in liver transplantation including occlusion of the superior vena cava (SVC), IVC and PV, as well as cold liver protection, fluid perfusion, liver IRI and passive intestinal congestion^27^,^28^,^29^. In addition, compared with allogenic OLT, this rat AOLT model has advantages such as avoiding transplant rejection, reproducibility, and high survival of rats.

It is known that BUN and SCr serum levels, as well as kidney pathological changes, could reflect kidney damage. Occlusion and open of the PV and IVC result in renal IRI; in which, cytokines enters the blood circulation and damage remote organs and systems. These cytokines could induce tubular epithelial cell necrosis and renal tubular atrophy; and further lead to renal function injury. In this current study, kidney function was found damaged in the M group, suggesting the presence of AKI induced by OALT. This study also demonstrated that this OALT model is suitable for research purposes.

As a member of the family of transmembrane proteins, TLR4 acts as a signal transduction molecule, which mainly expresses in human kidneys, myocardial cells, enterocytes, etc^30^. TLR4 uses four adaptors, including MyD88, TIRAP, TRIF and TRAM. TLR4-mediated signaling pathways mainly stimulate NF-κB activation^31^. The signaling of TLR4 is largely divided into two pathways: MyD88-dependent and TRIF-dependent which commonly activate the canonical NF-kB pathway for the induction of inflammatory cytokines. The NF-κB heterodimer consists of p50 and p65 (Rel A) subunits and it is a nuclear transcription factor that recognizes a common consensus DNA sequence which regulates a large number of target genes. It plays a pivotal role in immune and inflammatory responses by regulating the expression of several proteins including pro-inflammatory cytokines, chemokines and adhesion molecules; which further activates the NF–κB pathway and aggravates renal injury from ischemia/reperfusion^11^. Recent studies have demonstrated that the TLR4/NF-κB pathway plays an important role in mediating deleterious effects in renal injury from ischemia/reperfusion^11^,^12^. A recent study by Wu *et al.*^13^ demonstrated that TLR4-deficient and adaptor molecule myD88-deficient mice were protected from both kidney dysfunction and histological damage induced by renal IRI. In this present study, a significant increase in renal TLR4 expression, NF-κB activity, as well as in IL-1β and TNF-α levels in kidney tissues, were observed in the M group, compared with the S group; indicating that renal TLR4/NF-κB pathway was activated and downstream cytokines were synthesized following OALT, which further led to renal inflammatory damage. In the current study, nuclear localization of p65 appear to present both in tubular epithelial cells, glomerular cells and interstitial cells as shown in Fig. 5. This finding is in line with previous studies^32^,^33^,^34^,^35^,^36^,^37^ which showed that upregulation of expression of cytokines or activation of NF-κB in glomeruli, renal tubules, and the peritubular interstitium were detected during the acute kidney injury induced by ischemia reperfusion injury. These suggest that interstitial cells (such as morphologies, resident leukocytes/monocytes) may also play an important role in the acute kidney injury, which deserved further study. In the future, we should consider to isolate the primary cells of each type of renal cells and explore their relative role in the development of acute kidney injury.

Dexmedetomidine has been shown to have an organ protection effect. Kocoglu *et al.*^16^ revealed that dexmedetomidine was effective in protecting against cardiac IRI in rats. Hoffman *et al.*^17^ found that dexmedetomidine has a neuroprotective effect in rats. Kocoglu *et al.*^18^ and Frumento *et al.*^19^ revealed that dexmedetomidine could alleviate renal IRI. Recent studies^38^,^39^,^40^ found that dexmedetomidine could exert potential protective anti-inflammatory effects by reducing inflammatory cytokine serum levels. However, its upstream mechanisms have not been clearly revealed^41^,^42^.

This current study revealed that dexmedetomidine pretreatment, especially in high doses, prevents OALT induced pathological and functional injury to kidneys. These results indicate that the renal protection of dexmedetomidine was dose-dependent, which were consistent with previous reports. We propose that dexmedetomidine decreases inflammatory responses in kidney tissues of OALT rats by suppressing the TLR4/NF-κB pathway. Interestingly, our results are consistent with a previous report conducted by Wu *et al.*^43^, which documented that dexmedetomidine downregulated TLR4/MyD88/NF-κB pathway in rat lung tissues in response to LPS, suggesting that TLR4/MyD88/NF-κB pathway may be the target of dexmedetomidine in conferring organ protection.

Previous studies have shown that α_2_ agonist-induced sedation, analgesia, hypotension and hypothermia are mediated by the α_2A_-subtype^44^. The α_2C_-subtype mediates startle reflex, stress response and locomotion. The hypertensive and peripheral hyperalgesic effect of norepinephrine is mediated by the α_2B_-subtype^45^. Dexmedetomidine is a highly specific α_2_-adrenoceptor agonist with high affinity to each of the α_2_-adrenoceptor subtypes. Previously, it has been revealed that the organ protective effect of Dexmedetomidine was mediated by the α_2A_-adrenoceptor subtype. Ma *et al.*^46^ and Paris *et al.*^39^ discovered that dexmedetomidine was neuroprotective, and that this action was mediated by the α_2A_-adrenoceptor subtype. Ibacache *et al.*^40^ revealed that dexmedetomidine had myocardial protective effects by stimulating the α_2C_-adrenoceptor subtype. In our present study, we focused on the classical inflammation pathway and we did find that Dex affected this pathway in OALT-induced AKI, which has been confirmed by using NRK-52E cell line to show dexmedetomidine directly suppress the TLR4/NF-κB pathway activation mostly via adrenoceptor alpha 2A receptor.

These findings should jointly support our concept/conclusion that inhibition of TLR4/MyD88/NF-κB pathway activation may represent a major mechanism whereby dexmedetomidine reduces acute kidney injury after orthotopic autologous liver transplantation. However, further study incorporating the use of gene knock-out mice for TLR4, MyD88 and/or NF-κB should be performed in order to confirm the role of TLR4/MyD88/NF-κB pathway activation in dexmedetomidine mediated attenuation of acute kidney injury after orthotopic autologous liver transplantation. There are some limitations in this current study. The effect of post-treatment with dexmedetomidine was not determined. In addition, TLR4 mRNA levels by PCR were not provided. Further research is needed to explore these areas.

## Conclusions

In summary, these results substantiated our hypothesis that inflammatory reaction triggered by fluctuating hemodynamic changes and severe liver IRI contributed to OALT-induced AKI. Dexmedetomidine pre-treatment resulted in a renal protective effect during OALT. The potential mechanism for this effect is through inhibiting renal inflammation by suppressing TLR4/NF-κB inflammatory factor pathway activation. This effect was mediated by activating the α_2A_-adrenergic receptor subtype. The properties of dexmedetomidine may decrease the mortality rate of OALT rats. It should be noted that dexmedetomidine has been proved to preserve renal blood flow in number of pathophysiological status^47^, which may have contributed, in part, to dexmedetomidine mediated attenuation of acute kidney injury after orthotopic autologous liver transplantation and deserves further study. However, the pathway of TLRs is complicated, and the mechanisms by which these observed results occur require further investigation.

## Methods

### Chemicals

Dexmedetomidine (Hengrui, Jiangsu, China), atipamezole (Sigma-Aldrich, USA), BRL44408 (Sigma-Aldrich, USA), and ARC239 (Santa Cruz, USA) were dissolved in 0.9% saline at 2.5 μg/ml, 25 μg/ml, 50 μg/ml and 2.5 μg/ml concentrations, respectively. Heparin (Chen Xin Pharmaceutical Co., Ltd., Shandong, China) was diluted in 0.9% saline or acetic acid Ringer’s solution at 25 U/ml; and 12.5 U/ml of protamine sulfate (KaiYue Pharmaceutical Co. Ltd., Beijing, China) was diluted in saline at 0.05% concentration.

### Animal preparation and groupings

Experimental protocols and design were approved by the Sun Yat-sen University Animal Experimentation Committee. Animal care was performed according to Sun Yat-sen University Guidelines for Animal Experimentation. Male Sprague–Dawley rats (250  ± 30 g) were obtained from Guangdong Medical Experimental Animal Center (Guangzhou, China). In all experiments, all rats were maintained under standard conditions (room temperature at 25–27 °C with 12-hour light/dark cycle) with free access to specific pathogen-free (SPF) laboratory diet and distilled water for one week before the start of the experiment. Food was withheld 12 hours before the start of experiments, but all animals had free access to water.

A total of 77 rats were randomized into 11 groups (*n* = 7 per group) using a random number table, taking into consideration the weight of rats.

1. Rats in the S group (sham-operated group) were subject to abdominal incision, vascular dissection and wound closure without hepatic vascular exclusion and perfusion.
2. Rats in the M group (model group, established OALT model) underwent OALT operation including portal vein (PV) and inferior vena cava (IVC) occlusion for 20 minutes, followed by reperfusion for eight hours.
3. Based on the previous studies^48^,^49^,^50^, rats in the D1 group (10 μg/kg of Dex + OALT group) received 10 μg/kg of Dex injected intraperitoneally (i.p.) 30 minutes prior to OALT.
4. Based on the previous studies^48^,^49^,^50^, rats in the D2 group (50 μg/kg of Dex + OALT group) received 50 μg/kg of Dex injected i.p. 30 minutes prior to OALT.
5. Based on the previous studie^38^,^48^,^49^,^50^, rats in the B1 group (atipamezole + OALT group) received atipamezole (a non-specific inhibitor of α_2_-adrenergic receptors) at 500 μg/kg injected i.p. 40 min prior to OALT, followed by 50 μg/kg of Dex injected i.p. 30 minutes prior to OALT.
6. Based on the previous studies^38^,^48^,^49^,^50^,^51^,^52^, rats in the B2 group (ARC-239 + OALT group) received ARC239 (a relative specific inhibitor of α_2B_-adrenergic receptors) at 50 μg/kg injected i.p. 40 minutes prior to I/R, followed by 50 μg/kg of Dex injected i.p. 30 minutes prior to OALT.
7. Based on the previous studies^38^,^48^,^49^,^50^,^51^,^52^, rats in the B3 group (BRL44408 + OALT group) received BRL44408 (a relatively specific inhibitor of α-2a-adrenergic receptors) at 1.5 mg/kg injected i.p. 40 minutes prior to OALT, followed by 50 μg/kg of Dex injected i.p. 30 minutes prior to OALT.
8. Rats in the C1 group received 50 μg/kg of Dex injected i.p.
9. Rats in the C2 group received 500 μg/kg of atipamezole injected i.p., followed by 50 μg/kg of Dex injected i.p.
10. Rats in the C3 group received 50 μg/kg of ARC239 injected i.p., followed by 50 μg/kg of Dex injected i.p.
11. Rats in the C4 group received 1.5 mg/kg of BRL44408 injected i.p., followed by 50 μg/kg of Dex injected i.p.

### Establishment of rat OALT model

Rats were anesthetized with isoflurane (The Third Affiliated Hospital of Sun Yat-sen University, Guangzhou, China). Then, rat OALT models were established as previously described by Chi *et al.*^53^, and modified by Yao *et al.*^54^. This model simulated the main surgical steps and pathophysiological course of human liver transplantation including blocking and unclamping of the hepatic artery and portal vein, liver IRI, intestinal congestion, and hypoxia.

Animals were sacrificed eight hours after reperfusion based on previous experiments in rats, which demonstrated that kidney injury was most serious eight hours after reperfusion^55^. Kidney tissues were removed for histopathological evaluation, Western blotting, immunofluorescence analysis, and ELISA; while blood samples were taken from the abdominal aorta eight hours after liver reperfusion for blood urea nitrogen (BUN) and serum creatinine (SCr) measurements.

### Cell Culture

NRK-52E cells (kidney tubular epithelial cells) were obtained from American Type Culture Collection (Manassas, VA) and cultured in Dulbecco’s Modified Eagle’s Medium/F-12 supplemented with 10% fetal bovine serum. Cells were grown at 37 °C in an atmosphere of 5% CO_2_ in air.

### H/R protocol

The H/R protocol was performed as previously described with modifications^56^. Briefly, for hypoxia, confluent cells were incubated for 24 hours in an anaerobic chamber equilibrated with 5% CO_2_ and 95% N_2_. A metal catalyzer (Engelhard, USA) was used to maintain a constant low oxygen concentration (<0.1%) in the chamber. After the hypoxia treatment, the cells were transferred back to a regular incubator with 21% oxygen for 4 hours.

### Dexmedetomidine and siRNA preparation, screening and deliver

NRK-52E cells were confluent and pretreated with Dex at clinically relevant concentrations (1 nM)^57^ for 1 h immediately followed by the H/R treatment. Three siRNA duplexes targeting adrenoceptor alpha 2A rat gene (GenBank accession ID: 25083) were designed using the siRNA Target Finder and Design Tool available at http://www.ambion.com and were commercially obtained from Biomics (Jiangsu, China). Green fluorescent protein (GFP) siRNA, with no homology to adrenoceptor alpha 2A gene, was taken as siRNA control. After transfection using Lipofectamine^®^ 3000 transfection reagent (Life technologies) and incubation for 48 hrs, NRK-52E cells were harvested and expression of adrenoceptor alpha 2A mRNA level was detected by real time PCR (RT-PCR). Adrenoceptor alpha 2A siRNA that had the maximum inhibition rate was selected in our *vitro* experiments.

Adrenoceptor alpha 2A siRNA1,

5′-CGAGCUGCAAGAUUAACGA dTdT-3′(sense),

3′-dTdT GCUCGACGUUCUAAUUGCU-5′(antisense);

Adrenoceptor alpha 2A siRNA2,

5′-CUGGUUAUUAUCGCAGUGU dTdT-3′(sense),

3′-dTdT GACCAAUAAUAGCGUCACA-5′(antisense);

Adrenoceptor alpha 2A siRNA3,

5′-CUUUGGCCAACGAGGUUAU dTdT-3′(sense),

3′-dTdT GAAACCGGUUGCUCCAAUA-5′(antisense).

### Lactate dehydrogenase (LDH) assay

Cells were seeded at low density (10,000 cells/cm^2^) in 96-well plates. At the end of different stimulation, LDH assays was carried out according to the manufacturer’s introduction (Roche Diagnostics, Indianapolis, USA) as described in the study of *Luo C et al.*^55^.

### Kidney histopathological evaluation

Kidney specimens were fixed in 10% buffered formalin and embedded in paraffin. Half of the paraffin-embedded kidney tissues were cut into 5-μm sections for histopathological analysis using hematoxylin–eosin staining. The remaining half of the kidney tissues and contralateral kidney cortex samples were immediately frozen in liquid nitrogen for protein quantitative assay and immunohistochemical analysis. Severity of the kidney injury was evaluated by two histologists who were initially blinded to the experiment. A semi-quantitative scale was used in evaluating the morphological characteristics of tubules as suggested by Hamer’s standard *et al.*^58^.

### Biochemical analysis

Serum was isolated from blood by centrifugation (1,500 rpm at 4 °C for 10 minutes). BUN and SCr were analyzed as indicators of impaired glomerular function with an automatic biochemistry analyzer (Watford Olympus AU640, UK), based on manufacturer’s instructions.

### TLR4 and NF-кB p65 analysis by Western blotting

Kidney tissue or NRK-52E cells were ground and homogenized with a protein lysis solution (KeyGen BioTech, China). Nuclear and cytoplasmic proteins were extracted by using nuclear and cytoplasmic extraction reagents according to the manufacture’s instructions (Keygen Biotech. Co., LTD, Nanjing, China). The samples were separated by sodium dodecyl sulfate polyacrylamide gel electrophoresis (SDS-PAGE) and electro-transferred onto a nitrocellulose membrane. Subsequently, the membrane was blocked with 5% non-fat milk for two hours at room temperature, followed by overnight incubation with the rabbit polyclonal anti-TLR4 antibody (1:2,000, Santa Cruz Biotechnology, USA), the rabbit polyclonal anti-nuclear factor-kappa B (NF-kB) p65 antibody (1:500, Santa Cruz, USA), the rabbit polyclonal anti-Histone H3A polyclonal antibody (1:500, Santa Cruz, USA) and the rabbit polyclonal anti-β-actin (1:500, Santa Cruz, USA). The membrane was then washed and incubated with a secondary antibody (goat anti-rabbit IgG, HRP-linked antibody 1:4,000, Cell Signaling Biotechnology, USA) for two hours. Blotted protein bands were visualized by enhanced chemiluminescence (ECL) Western blotting detection reagents (Amersham, USA). Protein density measurement was correlated to protein expressions, and normalized with respect to Histone H2A or β-actin band density.

### Immunofluorescence analysis for NF-кB p65

NF-кB p65 in rat kidney was detected according to manufacturer’s instructions. Paraffin-embedded kidney sections were dewaxed and rehydrated. After three hours of antigen retrieval in 10 mM of sodium citrate (pH 6.0), the sections were incubated with blocking buffer (5% BSA in PBS) for one hour at room temperature; then, stained with NF-κB p65 (1:1,000, Santa Cruz Biotechnology, USA) at 4 °C overnight, followed by anti-rabbit IgG (1:1,000). To identify nuclei, tissues were counterstained with fluorescent dye DAPI for three minutes. In all cases, antibody negative controls were evaluated to ensure that results were not a consequence of cross-reactivity or non-specific binding of secondary antibodies. All images were measured using a Leica DM 4000 B scanning confocal microscope (Leica, USA). These sections were observed under a light microscope by an investigator who was initially blinded to the treatment groups, and 10 randomly selected fields of each slide were semi-quantified.

### Enzyme-linked immunosorbent assay (ELISA) of cytokines

100 mg wet kidney tissues were prepared as 10% tissue homogenates, and centrifuged at 3000 rpm at 4 °C for 10 min. The supernatant was collected for further analysis. The concentrations of TNF-α and IL-lβ in kidney tissue or in the cell culture medium were measured according to manufacturer’s instructions using ELISA kits (Nanjing Keygen Biotech Co., Ltd.). Absorbance at 450 nm (OD 450) was determined using a microplate reader.

### Statistical analysis

All statistical analyses were performed with SPSS Version 12.0 (SPSS Inc., Chicago, IL, USA). Quantitative data are presented as mean ± standard deviation (SD). Multiple comparisons among groups were analyzed using repeated measures by one-way analysis of variance (ANOVA) when the datas were normally distributed and the variances of the groups are homogeneous. Otherwise, Wil coxon Rank-Sum test should be used. Least significant difference (LSD) was used to assess differences between groups. Chi-square test was carried out on count data. A two-tailed *P* value less than 0.05 was considered statistically and significantly different.

## Additional Information

**How to cite this article**: Yao, H. *et al.* Dexmedetomidine Inhibits TLR4/NF-κB Activation and Reduces Acute Kidney Injury after Orthotopic Autologous Liver Transplantation in Rats. *Sci. Rep.* **5**, 16849; doi: 10.1038/srep16849 (2015).

## Figures and Tables

**Table 1:** weight and time of anhepatic phase between any of the groups.

Group	Weight (g)	Time of anhepatic phase (minute)
S group	241.3 ± 18.1	–
M group	240.6 ± 15.0	19.9 ± 0.5
D1 group	234.1 ± 20.3	19.8 ± 0.8
D2 group	240.5 ± 16.8	20.0 ± 0.6
B1 group	243.4 ± 21.6	20.0 ± 0.7
B2 group	243.1 ± 17.5	20.1 ± 0.6
B3 group	239.9 ± 21.0	20.2 ± 0.5
C1 group	242.6 ± 13.0	–
C2 group	240.3 ± 16.5	–
C3 group	241.4 ± 21.6	–
C4 group	237.9 ± 21.0	–

Weight and time of anhepatic phase (*n* = 7, 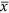 ± SD). There were no significant differences in weight and time of anhepatic phase.
